# A Cow–Calf Farming System Fully Adapted to Elevation and Harsh Conditions in Andorra (Europe)

**DOI:** 10.3390/ani11030611

**Published:** 2021-02-26

**Authors:** Ramon Armengol, Marta Bassols, Lorenzo Fraile

**Affiliations:** 1 Department of Animal Science, ETSEA, University of Lleida, 25198 Lleida, Spain; ramon.armengol@udl.cat (R.A.); marta.bassols@udl.cat (M.B.); 2 Agrotecnio Research Center, ETSEA, University of Lleida, 25198 Lleida, Spain

**Keywords:** ruminants, beef cattle, extensive production, local breed, Bruna d’Andorra

## Abstract

**Simple Summary:**

Optimizing the use of natural resources in cattle production is a concern of the beef meat industry and consumers. Areas with scarce resources or adverse environmental conditions can efficiently take advantage of extensive systems with local breeds. These local breeds are adapted to the environment and can give optimal productive yields. The aim of this work is to explain the project of the Bruna d’Andorra, a local breed under an extensive cow–calf system in Andorra, as an example of local farming and marketing of its meat products. Andorra is a sovereign landlocked microstate in the eastern Pyrenees (Europe) that consists predominantly of rugged mountains and harsh weather conditions for livestock farming. This work describes the evolution of the Bruna d’Andorra population during the period of 2000–2020 focusing on the main meat productive and reproductive performance achievements. Finally, the plans for the future of the project are focused on optimizing a highly sustainable and environmentally friendly farming system.

**Abstract:**

The use of natural resources is an important topic to optimize the efficiency of cattle production. The purpose of this work is to describe the project of the Bruna d’Andorra; a local cow breed under an extensive cow–calf system in Andorra (Europe), as an example of local farming and marketing of its meat products in an area with adverse environmental conditions. This breed is located in Andorra, a microstate that consists predominantly of rugged mountains and harsh weather conditions. The cow–calf Bruna d’Andorra extensive system is thoroughly described and productive and reproductive performance, compiled over 21 years (2000–2020), has been analyzed by years with the Chi-square test or ANOVA to compare proportions or means, respectively, and regression analysis was used to decipher evolution across years. The results show a population with a census large and stable enough to avoid inbreeding. Moreover, a sustained improvement of the productive performance and maternal fitness has been observed along the studied period for Bruna d’Andorra. The work concludes that local breeds can achieve sustainable animal production, especially when farmers, public administration and commercial circuits in the area agree to cooperate on such projects. The study also concludes that the Bruna d’Andorra cow breed can still improve in meat and reproductive performance.

## 1. Introduction

Mountain farming livestock has attracted a wide research worldwide in the past 30 years. These pasture-based and small-scale livestock farming systems can be considered as the main source of livelihood in the mountain primary sector, ensuring socioeconomic sustainability and biodiversity in rural communities [1,2]. For the last 25 years, scientific research has been carried out in areas close to Andorra with cattle breeds adapted to high mountain pastures, such as Bruna dels Pirineus (BP), Parda Alpina (PA), Gasconne (G), Aubrac (A), Limousin (L) or Salers (S) in order to improve their genetics, productivity or meat quality [3,4,5,6,7].

**Figure 1:**
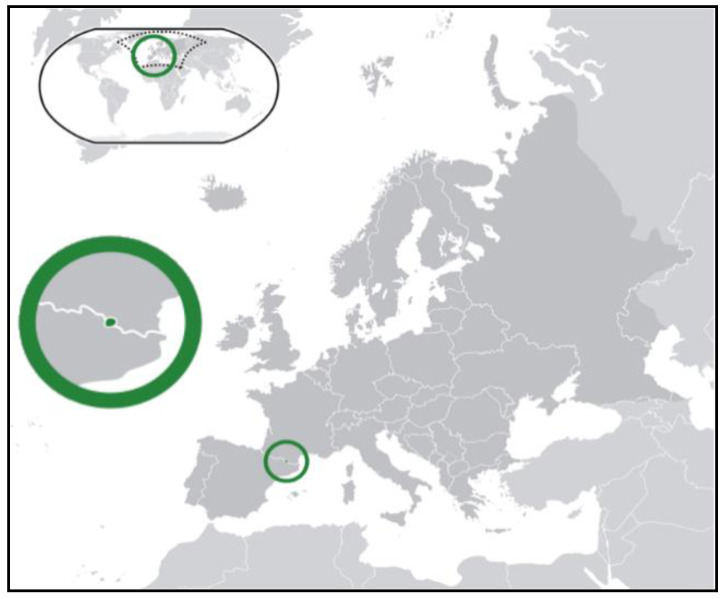
Location of Andorra (green circle) in Europe (dark grey). Source: https://en.wikipedia.org/wiki/File:Location_Andorra_Europe.png. (Accessed on 29 September 2020).

Andorra, officially Principat d’Andorra (Principality of Andorra), is a sovereign landlocked microstate on the Iberian Peninsula in the eastern Pyrenees that is bordered by France to the north and Spain to the south (Figure 1). It has an area of 468 km^2^ and a population of approximately 77,000. Andorra la Vella, its capital, has the highest elevation of any European capital at 1023 m. Andorra’s territory is divided in seven parishes, consists predominantly of rugged mountains, and the average elevation is 1996 m. Andorra has alpine, continental, and oceanic climates. The climate depends on elevation. The climate is characterized by convective and abundant rains during the spring and summer, which can last until autumn. Winter, however, is characterized by a great amount of snowfall. The temperature regime is broadly characterized by a temperate summer and a long and cold winter, which is in keeping with the mountainous terrain of the country [8].

Traditionally, Andorra based its economy on pasture and small-scale livestock farming systems that mainly included sheep, horses and cattle. However, since the 1950s, the primary sector has been losing importance. Nowadays, the tertiary sector currently represents 80% of Andorran Gross Domestic Product (GDP), with tourism being the mainstay of the economy. Eight to nine million people visit it every year, attracted by its status as a tax heaven, its ski resorts, and the trade price differential with neighboring countries [9]. Only five percent of the land is arable, and tobacco is the main crop. Farming is based on an extensive and semi-extensive pasture system with a clear and ancestral purpose: the subsistence of its population in one of the most mountainous and remote areas of Europe (three aptitudes: work, meat and milk). With the entry into the modern era of the animal production system (~1950s), bovine livestock became a single goal, which was the breeding of calves, with an extensive and semi-extensive cow–calf farming system. These calves were weaned at 5–6 months of age and sold to Spanish producers, where they finished their conventional fattening phase. The calves were slaughtered in Spain and the meat was fully marketed in conventional Spanish circuits.

Until year 2000, the breeds that made up the bovine population of Andorra were purebreds and crosses of BP, L, G, A and Charolais. Breed selection, crossbreeding, and bovine candidates to become cows and bulls only depended on the farmers’ subjective criteria, and it was never based on technical data. Thus, the diversity in the final product varied widely and the production parameters were not controlled.

In 1999, the national association (Associació de Pagesos i Ramaders d’Andorra) (APRA) [10] and the Government of Andorra (GA) joined forces to create Ramaders d’Andorra, S.A. (RASA) with a common long-term project named Carn de Qualitat Controlada d’Andorra (Quality Control Meat of Andorra) (CQCA) and registered the label. In 2000, the GA approved a new law of agriculture and livestock and promoted the creation of the Escorxador Nacional d’Andorra (National Slaughterhouse of Andorra) (ENA) following the European Union (EU) regulations. In 2006, after an initial work of breed selection and technical criteria, an official rule issued by the GA created the Genealogical Card for Bruna d’Andorra Bovine Breed (BA), set the technical criteria for genetics of this breed and the staff responsible for this program [11]. In 2009, the GA applied for the “IGP Carn d’Andorra” label in the EU, describing that the meat products included in the label must be from pure BA breed or BA cow crossed with other beef breeds. The label “IGP Carn d’Andorra” was finally accepted and registered in 2009.

The BA breed is genetically very closely related to the BP, an autochthonous beef breed located in the mountainous areas of Catalonia (Northeastern Spain) and PA, an autochthonous beef breed located in the mountainous areas of Aragon (Northeastern Spain). The BP and PA breeds originated from the cross of native cattle with imported old-type Brown Swiss individuals during the first decades of the 20th century. After that, animals were empirically selected for meat production purposes. BP and PA are typically reared under extensive conditions for the production of beef calves with an average carcass weight at slaughter of 330 kg at ~12.5 months of age [5,12].

The CQCA project and IGP Carn d’Andorra are based on the following pillars: unification of the BA breed, genetic improvement, health improvement, maximum use of the country’s natural resources, and full internal marketing of the meat. The main goal of this strategic project of national interest is to achieve a homogeneous bovine population of a typical local breed from Andorra, well adapted to the mountain pastures of the country, and able to maximize the use of natural resources in a sustainable way. This system of cattle production includes the breeding and fattening of calves, obtaining replacement animals, the slaughter of animals, and the marketing of their high-quality meat in the Andorran territory. The project includes BA cows and bulls, L bulls as breeders, and BA or crossed Bruna d’Andorra × Limousin (BA × L) males and females for fattening.

The aim of this work is to reflect the evolution of BA population, meat productive performance, and main reproductive data that have been achieved during this 21-year period (2000–2020). A second objective is to explain the plans for the future of the CQCA project as a highly sustainable and environmentally friendly farming system.

## 2. Material and Methods

Ethical Statement

All the data used in this study came from historical data using standard production procedures.

### 2.1. Cow–Calf System in Andorra

**Figure 2:**
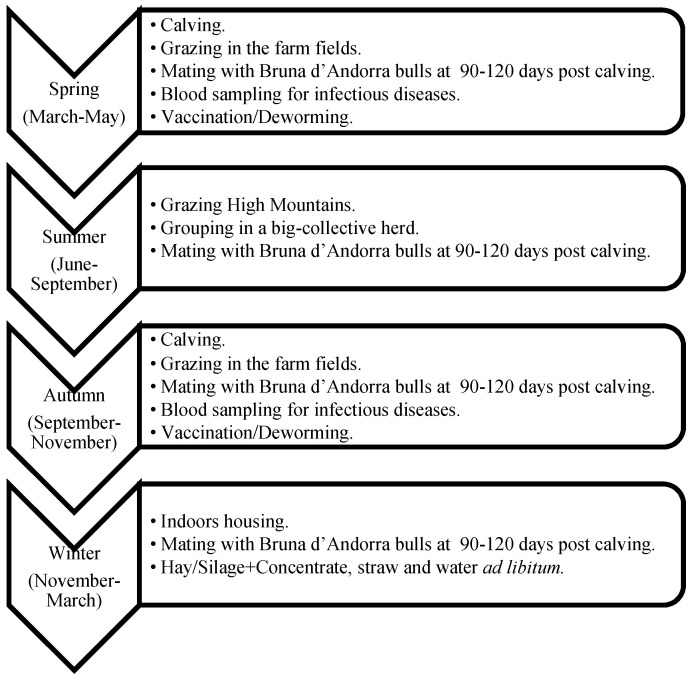
Management, nutritional, and husbandry protocols used with adult cows (>1 calving).

**Figure 3:**
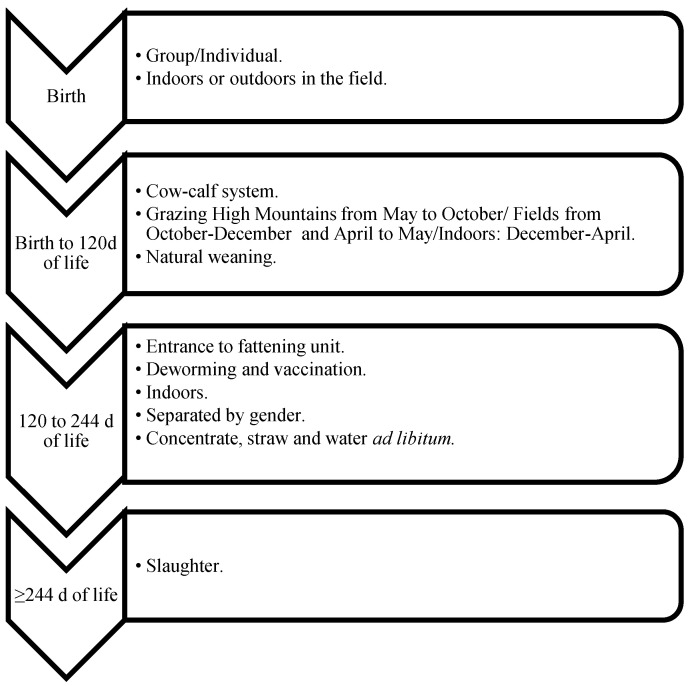
Management, nutritional, and husbandry protocols used during the calf-rearing process of Bruna d’Andorra Bovine Breed (BA) and Bruna d’Andorra × Limousin (BA × L) males and females destined for fattening.

**Figure 4:**
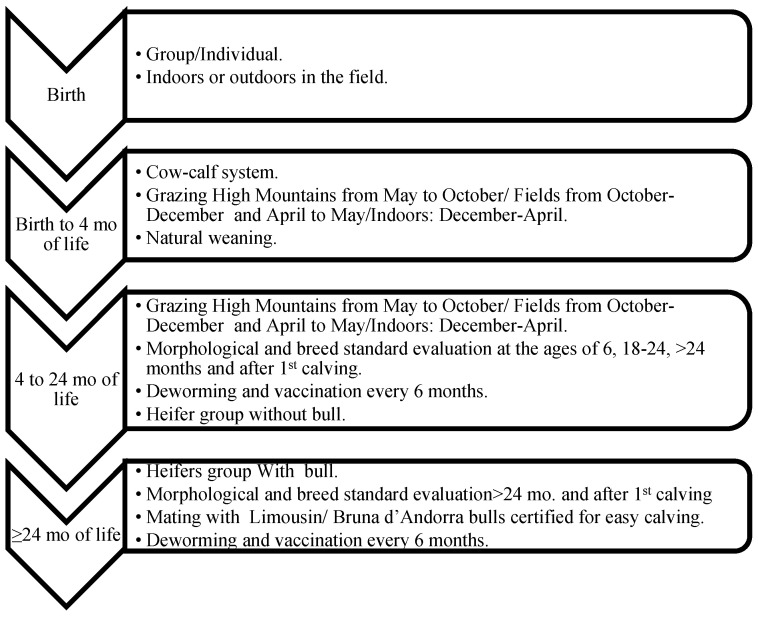
Management, nutritional, and husbandry protocols used during the calf and heifer rearing process of BA females destined for cow replacement.

**Figure 5:**
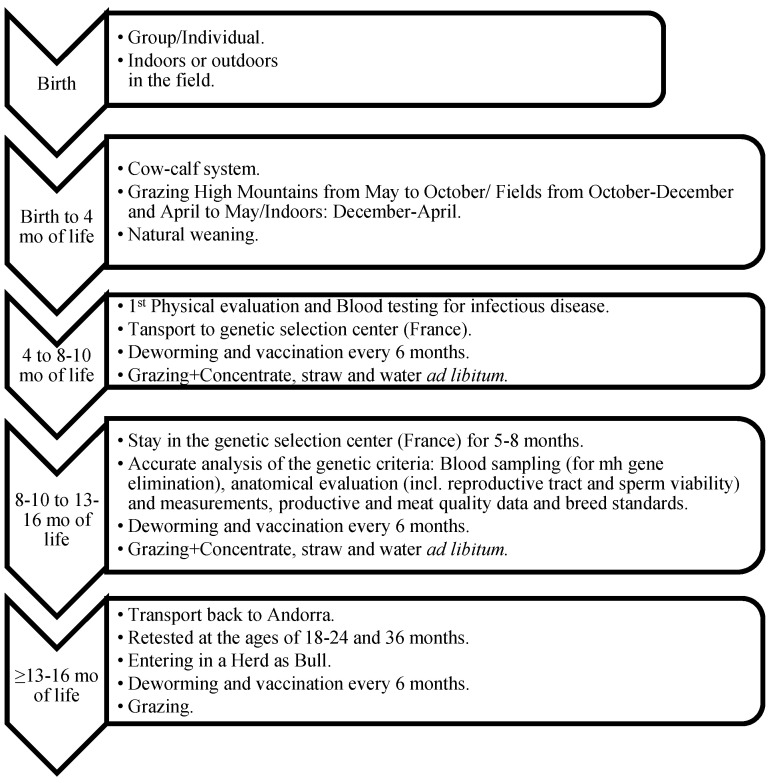
Management, nutritional, and husbandry protocols used during the calf and bull rearing process of BA males destined for bull replacement.

The traditional farming system in Andorra is extensive and semi-extensive cow–calf grazing, under mating as the only reproduction strategy, and a long pre weaning period (~4–6 months). After a cold and snowy winter (the end of November to the beginning of March) where all bovines are housed indoors down in the villages, all the bovines are set free to the fields nearby the farms and calving season begins (March–June). Animals can be moved indoors nightly depending on the weather conditions, and cows close to parturition are kept in the farm. After June, all animals from all farms that belong to the same parish are grouped, forming a big herd of approximately 200 to 400 mature cows, calves, and bulls each. This big herd will be grazing and pasturing the high mountains of Andorra under the supervision of a *vaquer* (cowboy in Catalan) and in cooperation with the farmers (owners). The *vaquer* is responsible for controlling the animals but also for deciding where to graze the herds, which depends on the weather and the vegetative state of the pastures. Therefore, *the vaquer* is a key figure in maximizing the resources of high mountain pastures. During this period, mating is possible. Adult cows and bulls are always together, with the goal to achieve a uniform distribution of calvings throughout the year. However, seasonality is always present, and two calving seasons are common: spring and autumn. In September–October, animals are sorted according to the farm they belong to and transferred to the fields close to the farm for grazing until the strong cold weather comes again. This system includes two important movements: the beginning and end of the high mountains grazing season. This movement is carried out by moving the animals through walk-through roads and trails, except for some farms that have to move the animals through the big city to get to the high mountains area or back to the farm. In these situations, part of the transport is by truck. During these movements, young bull selection takes place, and the ones selected are sent to the genetic center. Animals are selected to enter the fattening units and are dewormed, vaccinated, have a blood sample taken, and heifers are evaluated for replacement (Figure 2, Figure 3, Figure 4 and Figure 5).

Autumn and spring pastures in the low attitude fields close to the farms were based on planted *Medicago sativa, Dactylus glomerata, Festuca arundinacea, Lolium perenne,* and *Trifolium ladino.* Some fields were harvested as hay and/or silage as feeding source during indoor winter season. High mountains pastures were composed on *Festuca airoide, Hieracium breviscapum, Carex curvula, Oreochloa disticha,* and *Leontodon pyrenaicus* in elevations between 2300 and 2800 m; *Festuca eskia, Festuca gautieria, Festuca nigrescens, Festuca ovina, Anthoxanthum odoratum, Achillea millefolium, Trifolium alpinum, Trifolium repens, Trifolium pratense, Koeleria vallesiana, Thymus serpyllum, Caluna Vulgaris, Lotus corniculatus, Meum athamanticum, Vicia Pyrenaica, Scutellaria alpina, Festuca paniculata, Asphodelus albus, Iris latifolia, Paradisea liliastrum, Dactylis glomerata, Avenula versicolor, Campanula scheuchzeri, Nardus stricta, Gentiana acaulis,* and *Trifolium alpinumin* were at elevations between 1600 and 2700 m. At elevations between 1400 and 2200 m, some natural grazing areas were forested and composed of *Pinus uncinata* and *Abies alba*. The proportion of each plant present in the pastures also varied on the soil composition, water sources, direct sunlight, and each year’s climatology. Production in dry matter (DM) in natural pastures in Andorra was estimated in 1000 to 3000 kg/ha/year [13,14].

Females and males destined for fattening were required to spend at least the first four months of life with their mother and could not be sent to slaughter before eight months of age [11]. They were kept in semi-open fattening units with at least 3 m^2^/animal and according to their body weight (BW), feeding was ad libitum based on natural concentrate (cereals, legume and oilseeds, minerals, and vitamins), with hay of the mountain grass as a fiber source, and water (Figure 3). Heifers destined for future replacement followed the same grazing system and movements as the adults, but in an independent group without bulls in order to avoid mating too young and the subsequent dystocia and mortality problems (Figure 4).

It is of highest interest for the GA and RASA to maintain this system as a main characteristic of the BA breeding system, because it is the most sustainable and environmentally friendly for the country. 

### 2.2. Co-Working Government Farmers

The project began in 2000 with the aim of producing a high-quality meat that is standardized and officially recognized. Since then, the GA and RASA cooperated to obtain an EU high quality meat label. Carn d’Andorra^®^ is a trademark of RASA that is marketed under the control and guarantee seals CQCA and under the protected geographical indication seal “IGP Carn d’Andorra” [15].

### 2.3. The Breed

**Figure 6:**
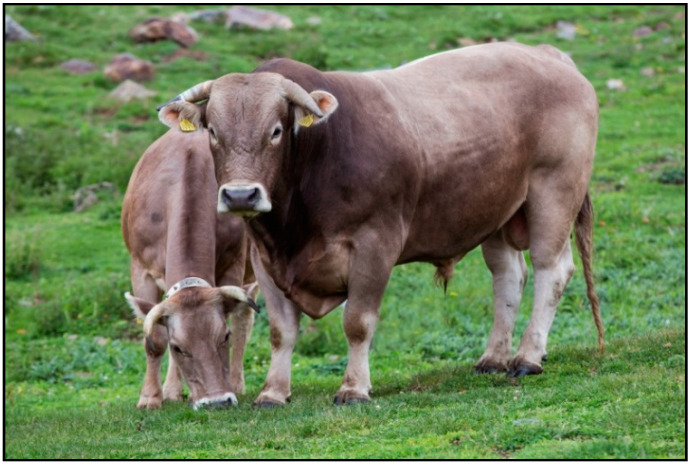
Adult female and male BA cattle. Source: Natàlia Montané.

The choice of the breed that was included in the CQCA project was the BP [16]. However, it was decided not to strictly follow the breed standards established by the BP Association (Catalonia) and to create the BA breed. This decision was for two reasons: to fully adapt the breed as much as possible to the Andorran productive system (adaptability to the high mountain environment and harsh weather conditions) and to create a breed with an Andorran identity name. This election was agreed by the GA and RASA, and officially adopted in 2006 [11]. Briefly, the BA is a cow very adapted at pasturing the high mountains (strong hooves and claws), obtaining food (wide nose and deep thorax) and feeding the natural vegetation of the area, is adapted to climatic difficulties and elevation (rain, snow, and low temperatures), has a marked maternal instinct, and calves with ease. These cows are brown, with color lightening in the eyes, nose, inside elbow, udder, belly, inner part of limbs, and perineum. There may be a degraded bar along the entire dorsal line, especially in males. Mucous membranes are pink to orange, and snouts are black. The hooves are evenly pigmented, round, and uniform. The average height is ~135 cm at shoulder and the thoracic perimeter is ~190 cm. The rusticity factors are enhanced: crest (especially in males), chin (may be sinuous, but not exaggerated), and thick limbs. The spine should describe a rectilinear profile and uniform. The head must be wide with a flat forehead, strong jaw, and well-drawn. The ears are big, with abundant white hair inside and eyes are big and vivid. Horns are in the shape of a low lyre and round section, whitish and with the black tip (Figure 6). The breed standards also consider of high interest the safety of humans (farmers and tourists) and animal welfare. Therefore, the selection program discards individuals aggressive to humans or any factor that can difficult calving ease (i.e., gen of muscular hypertrophy—Mh). The project also permits mating for the first calving with BA bulls negative to Mh (genetic profile: +/+) or L, in order to reduce dystocia, culling, and calf mortality risks. BAxL calves must be always destined for fattening and are not allowed to become breeders.

### 2.4. Genetic Improvement of the Breed

The BA genetic improvement program focused its efforts on maximizing the strengths of the breed (adaptability to the environment, acceptable productive yields, and good maternal fitness) and minimizing the weak, unwanted, or risky points (gene of Mh, inbreeding, and aggressiveness). The first step and most important point of the program was the selection of the bulls. At the age of eight to 10 months, male calves that were destined to be future bulls were selected as candidates in Andorra and transported to the Genetic Selection UPRA Gasconne Center (Villeneuve de Paréage, France). Trained technicians and veterinarians of the GA carried out this first selection. These males spent five to six months in the center for an accurate analysis of the genetic criteria. The genetic selection included blood sampling (for Mh gene elimination), anatomical evaluation (absence of defects, including genital tract and sperm viability–quality and quantity) and measurements (height at hock, body length—back, loin and rump—width at shoulders, width and depth at inside elbows, girth of chest, pelvic length and width, sacral-pubis bones length), productive (average daily gain—ADG) and carcass quality data (muscle and bones development), and breed standards. Based on all these measurements, bulls received a final qualification over 100 points. At the age of 14–16 months, these males returned to Andorra as BA bulls certified as breeders if they had successfully passed the test with ≥61 points (Figure 5). The genetic data compiled from the bull itself recommended/allowed the bull to remain in the herd where it was born or to be sold/transferred to another herd in order to avoid inbreeding. Bulls were retested at the ages of 18–24 and 36 months. 

Female calves, destined as future BA cows, never exited Andorra and their genetic selection was based on anatomical (absence of defects), morphological (rump and pin bones for maternal aptitude and meat yield performance parts of best interest) and breed standard evaluation at the ages of 6, 18–24, >24 months, and after their first calving. For safety and management reasons, the evaluation of the females was visual while pasturing and it was carried out by trained technicians and veterinarians of the GA. To be part of the selected BA cows, they were required to obtain 61 points out of 100 in the test.

In 2004, the BA breed national contest was created. Every 27 October, the best individuals in the evaluation of the BA breed received a national award in the different age categories. Another annual national event started in 2012: the certified BA bull auction in order to promote the transfer and purchase of the tested and certified BA bulls. These two events are considered to be of national interest and the team responsible for the project takes advantage of those events to explain the breeding and fattening system to the population and promote the meat consumption of BA meat under the label Carn d’Andorra^®^.

### 2.5. Health Program

Bovines in Andorra are not reared under bio/eco veterinarian and farming rules. However, it is of high interest to all the professionals in the project to feed the animals as naturally as possible, to reduce the use of antibiotics, and to control and eradicate diseases. Bovines from Andorra are free of Brucellosis and have low prevalence (<10%) of Infectious Bovine Rhinotracheitis (IBR), Tuberculosis, *Besnoitia besnoiti* (GA Veterinarian Service internal data), and *Neospora caninum* [17]. 

Bovines older than 180 days received parenteral deworming every six months. Official Veterinary Services consider the risk of parasitism to be high along all the seasons, as cattle share pastures with other domestic (sheep, goats and horses) and wild species (Roe deer (*Capreolus capreolus*), wild boars (*Sus scrofa*), chamois (*Rupicapra rupicara*) and mouflons (*Ovis orientalis musimon*). Anthelmintic drugs used (Moxidectin 0.2 mg/kg BW SC CYDECTIN^®^ Zoetis Spain, S.L. Alcobendas Spain; Ivermectin 0.2 mg/kg BW SC NOROMECTIN^®^, Norbrook Laboratories, Ireland or Doramectin 0.2 mg/kg BW SC DECTOMAX^®^. Zoetis Spain, S.L. Alcobendas Spain) are rotated taking into account the regular (yearly) parasite diagnosis in order to minimize the development of resistance under veterinary supervision. Pregnant cows are vaccinated against *Escherichia coli*, rota-, and coronavirus in order to prevent neonatal calf diarrhea (Rotavec^®^, Merck Sharp and Dohme Animal Health, S.L., Burgwedel, Germany). Animals destined to the fattening units are parenterally dewormed and vaccinated against *Clostridium spp.* (Covexin 10^®^, Zoetis Spain, S.L. Alcobendas Spain) at entry in the unit. 

Calving ease is reported by the farmer to the official database managed by the GA. Calving ease is scored CE0 to CE4 (CE0, easy; CE1, slight assistance; CE2, moderate assistance; CE3, difficult calving; CE4, extreme difficulty calving, cesarean, or fetotomy). Scores CE3 and CE4 are considered dystocia.

### 2.6. Slaughter and Meat Products

Since the beginning of the project, both the GA and RASA knew that to optimize the sustainability of the final product, the meat had to be processed, sold, and consumed as close as possible from the grazing areas. Thus, the processing and consumption had to be, ideally, within the Andorran territory. That is why the ENA was built in 2000 and the Andorran butchers and restaurants were asked to cooperate, taking part in the project and promoting the consumption of “IGP Carn d’Andorra” meat by the Andorrans and the large number of tourists that visit the country every year.

### 2.7. Data Compilation and Statistical Analysis

The data used for this descriptive study were from official sources of the GA, the RASA, and the ENA. During the study period, these three institutions manage databases with more than 20,000 data points from 47 farms. Data under study are: census (females > 12 months old, bulls, and animals in the fattening units), fattening performance (body weight—BW—at birth (kg), BW at slaughter (kg), average daily gain (ADG) (g/days of life, % meat yield (Kg carcass/kg BW ×100-)), carcass quality based on muscular profile with 6 rates: S (superior), E (excellent), U (very good), R (good), O (fair), P (poor) (SEUROP rating) [18], and reproductive performance (age at first calving, average number of calving, calving ease, interval calving–calving). Data compiled are from pure BA and BAxL breeds.

All statistical analyses were carried out using SAS V.9.1.3 (SAS institute Inc, Cary, NC, USA). For all analyses, the individual animal was used as the study unit. The significance level (*p*) was set at 0.05. The variables included in the statistical analyses were classified as nominal (carcass quality (SEUROP rating), gender, and breed (BA and BAxL breeds)), ordinal (calving ease and year) or continuous: body weight—BW—at birth (kg), BW at slaughter (kg), % meat yield (Kg carcass/kg BW ×100), age at first calving (months), average number of calvings, and interval calving–calving (months). Shapiro Wilk´s and Levene tests were used to evaluate the normality of the distribution of the continuous variables and the homogeneity of variances, respectively.

Contingency tables (Chi-square or Fisher exact tests) were used when the association between nominal and ordinal variables was assessed. To study the association between nominal or ordinal variables with the continuous non-normally distributed variables, the Wilcoxon test (with the Mann–Whitney U test to compare each pair of values) was used. To analyze the association between continuous normally distributed variables and nominal or ordinal variables, an ANOVA test (with Student´s t-test to compare each pair of values) was used. Finally, a Pearson or Spearman regression analysis was carried out to decipher the evolution of the year (as a continuous variable) with other normally distributed continuous variables or not, respectively. 

## 3. Results and Discussion

The data available cover a total of 21 years (2000–2020). During this period, the yearly population of bovines older than 12 months (cows, bulls, and replacements) ranged between 1008 and 1390 bovines. These data agree with the calculations that the government has for a sustained cow–calf production system considering the feeding yield in high mountain grazing areas. The data analyzed were from 14,128 calvings and 9982 slaughtered males and females, including pure BA and BAxL bovines. The complete data of this study are in the Supplementary Materials for interested readers (Tables S1 and S2).

### 3.1. Bruna d’Andorra Population Census

**Figure 7:**
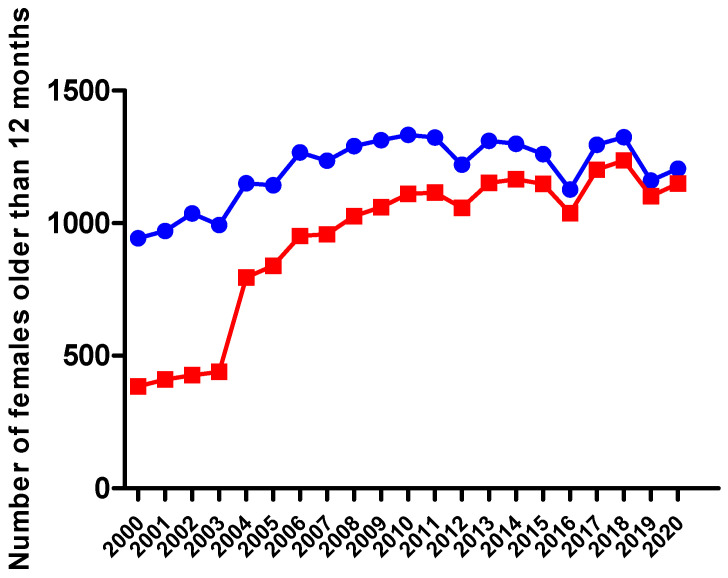
Evolution of total (blue filled circles) and BA females (red filled squares) older than 12 months from 2000 to 2020 in Andorra.

The number and proportion of BA bovines older than 12 months has always increased every year in a steady and controlled pace. In 2000, Andorra had a total of 384 females and nine bulls selected for the breeding program, corresponding to 39.7% of the bovine population of the country. In 2020, the population older than 12 months and considered for reproductive aptitude consisted of 95.4% of BA breed bovines, 1149 females and 54 bulls (see Figure 7 and Table S1 for yearly detailed data in Supplementary Materials). It is important to remark that farmers were allowed to introduce cows of the BP breed because it was a genetically closely related breed and there was a high risk of inbreeding at the beginning of the program. Since 2004, it is forbidden to introduce animals in the genetic program that are not born from a BA cow and bull. Other breeds that complete the adult bovine population in Andorra are G, A and L.

Between 2005 and 2010, there was an increase in the overall BA bovines that first indicated the possibility of maintaining the breed census and avoiding inbreeding problems. During these years, the fattening of BA increased compared to the previous years, showing that the genetic selection could be carried out with the best cows and bulls. It is of special interest to remark that between 2000 and 2004, any BA female entered to the fattening units and the number of BA bulls not selected as breeders and destined to fattening was always between zero and seven per year between 2000 and 2006. The last period under study (2011 to 2020) shows a sudden increase in the BA females and males destined to fattening per year (>125 and >220, respectively) as a consequence of the increase in BA calves born. This increase in BA animals in the fattening units relates to a decrease in BAxL animals, with less than 100 females and males per year since 2013 and 2015 (for yearly results see Table S1 in Supplementary Materials). This change in the fattening units breed composition is mainly because of the better calving ease of BA cows and heifers and the elimination of the Mh gene in the BA bulls allows the farmers to decide that the first calving can be the pure BA breed without risk. Another reason is that farmers prioritize the chance to increase the selection of future BA cows than fatten crossed animals with better meat yield. Table S1 in Supplementary Materials shows the yearly decrease of L bulls along the period under study. To sum up, the project began with 18 to 27 L bulls (year 2000 to 2007) and this population is under 10 bulls since 2013. It is important to remark that in years 2019 and 2020, only one L bull was destined for breeding.

### 3.2. Productive Improvement of the Breed

The increase in BA breeder census (heifers, cows, and bulls) guarantees the viability of the genetic program. As a result, some productive parameters such as calving ease (CE0–CE4), average daily gain (g BW/day), meat yield (%), and carcass quality (SEUROP) have all improved over these years. Heifers and young bulls discarded for replacement, removed adult cows and bulls were also introduced in the market as CQCA.

**Figure 8:**
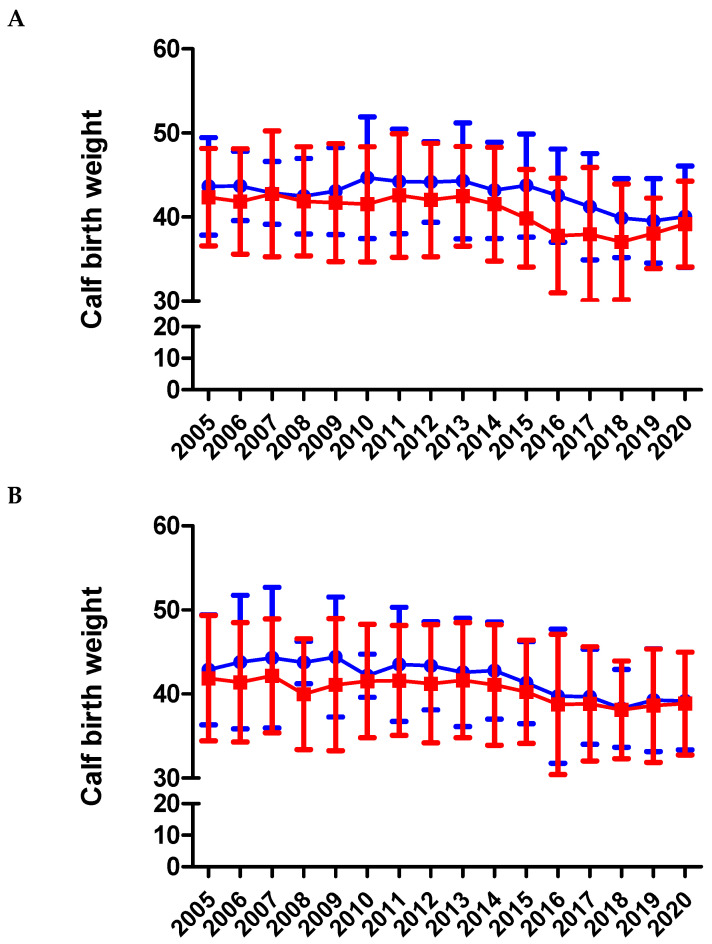
Birth weight of calves from BA (**A**) and BAxL (**B**) split by males (blue filled circles) and females (red filled squares) from 2005 to 2020 in Andorra. Calf birth weights decreased linearly across years for both genders and breed groups (*p* < 0.0001); and bull calves were consistently heavier than heifer calves across breed groups (*p* < 0.001).

The compilation of BW at birth began in 2005. During the whole period studied, males from both breeds under study (BA and BAxL) had a significantly higher BW than females. The average BW was 40.2 ± 4.5 kg for BA males, 42.2 ± 0.8 for BAxL males, 39.2 ± 4.3 kg for BA females, and 40.6 ± 0.8 kg for BAxL females (*p* < 0.0001). BW has gradually and significantly decreased along all years in both genders and for both BA and the BAxL breeds (*p* < 0.001) (Figure 8A,B). At the beginning of the program (2006–2010), BA female calves averaged ~42 Kg (always over 41 Kg) and since 2016, the BW has always been below 40 kg. In BA male calves, the average BW has decreased irregularly, but decreased from ~43–44 (years 2006–2010) to ~39–40 kg (2016~2020) (For detailed yearly data see Table S2 in Supplementary Materials). In this sense, today’s BA breed calves seem to be lighter than calves from other genetically close related breeds such as BP (44.27 to 46.98 kg BW) [19,20] or PA and Brown Swiss (BS) (~45 Kg BW) [12,21]. Regarding other European breeds, adapted to pasture-based systems, and also common in the Pyrenees mountains, BA calves are lighter than G, with a BW at birth of ~45.3–40.7 Kg [22], but heavier than S or L, with a ~31.8–30.7 and ~28.8–33.5 Kg BW, respectively [23,24]. The authors highlight that the BW reported are from the year 2000 to 2014 for other breeds, so they might have also varied their BW at birth in the last six years. However, we could not find more references. For crossbred BAxL calves, BW has also decreased and has been generally below the pure breed calves. In BAxL female calves, BW decreased from ~41 kg to ~38 since 2016; for BAxL males, the decrease has been from ~42–44 Kg in period 2006–2010 to ~40 kg (see Table S2 for yearly detailed data in Supplementary Materials). On the other hand, it has been known for years that crossbreeding with L can improve calving ease and performance [25]. Thus, the well-known calving ease of the cross breeding with L bulls, the sturdiness, the good productivity, and the good performance, in terms of meat yield and carcass classification, at any age at slaughter [26] caused the decision to include the crossbreeding with L for first parturition BA heifers, but the main goal was to reduce the mortality and dystocia of BA first calving cows. The use of L bulls for crossbreeding in different countries and with dairy breeds has also demonstrated a higher ADG and live weight gain per kg of food consumed than those of any other breed [26].

**Figure 9:**
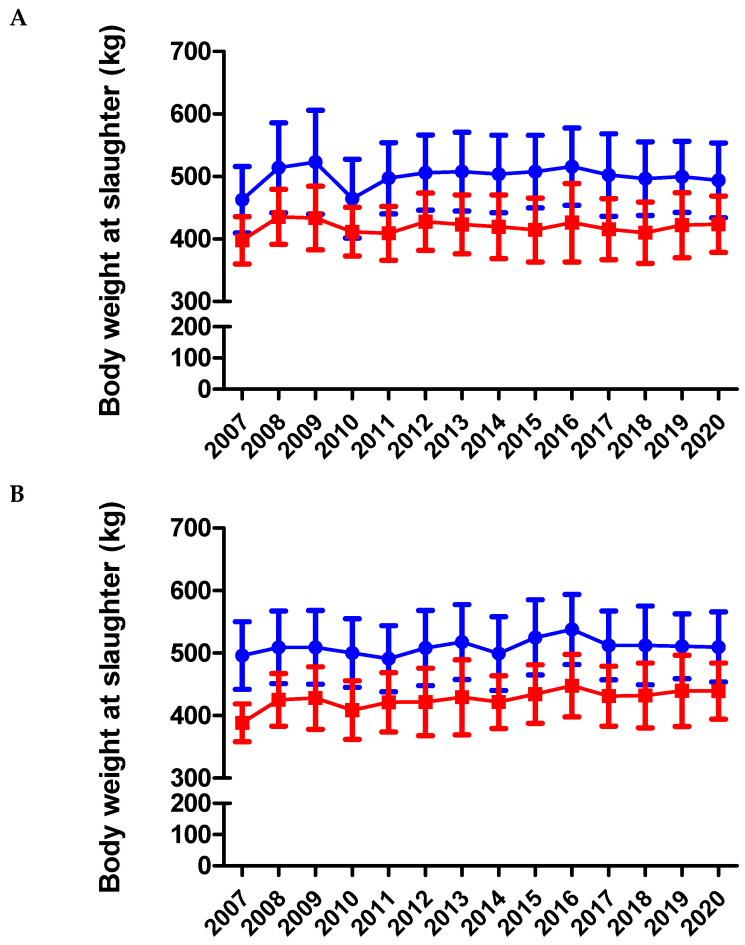
Body weight at slaughter from BA (**A**) and BAxL (**B**) split by males (blue filled circles) and females (red filled squares) from 2007 to 2020 in Andorra. Males had higher BW at slaughter than females in both BA and BAxL groups (*p* < 0.001) without observing a significant trend across the study period (*p* > 0.05).

As expected, males had a significantly higher BW at slaughter than females in both BA (504 ± 61 vs. 420 ± 51 kg) and BAxL (507 ± 59 vs. 425 ± 50 kg) groups (*p* < 0.001) (Figure 9A,B). As an overall result, the age at slaughter is ~365 days for all the animals sent to slaughter. The age and BW at slaughter for females and males of BA and BAxL have shown significant differences between the years under study (*p* < 0.001), but these differences do not show a consistent trend along the time (see Figure 9 and Table S2 for yearly results in Supplementary Materials). Of special interest is the significantly greater age and BW at slaughter of the BA compared to the BAxL between years 2005 to 2007 (Table S2 in Supplementary Materials). These differences are probably due to the fact that it was the first period where the genetic program could discard candidates as future cows and bulls. Thus, after testing and retesting, discarded animals were slaughtered with older ages. The inclusion of these animal data in the slaughtered BA animals has increased the BW at slaughter in this period of time. These between-year differences might be also due to the meat price variation, variability on the feeding cost, and the demand of product depending on market demand. It is important to remark that thousands of tourists visit Andorra, and a priority of the project is to always have CQCA/IGP Carn d’Andorra products to cover the market demand. The total number of visitors to the country is strongly influenced by snow conditions in winter, good weather in summer, and even on which day of the week the holidays are in neighboring countries (France and Spain). All of these factors have an effect on the demand for meat, especially in hotels and restaurants in Andorra, and are different between years. 

**Figure 10:**
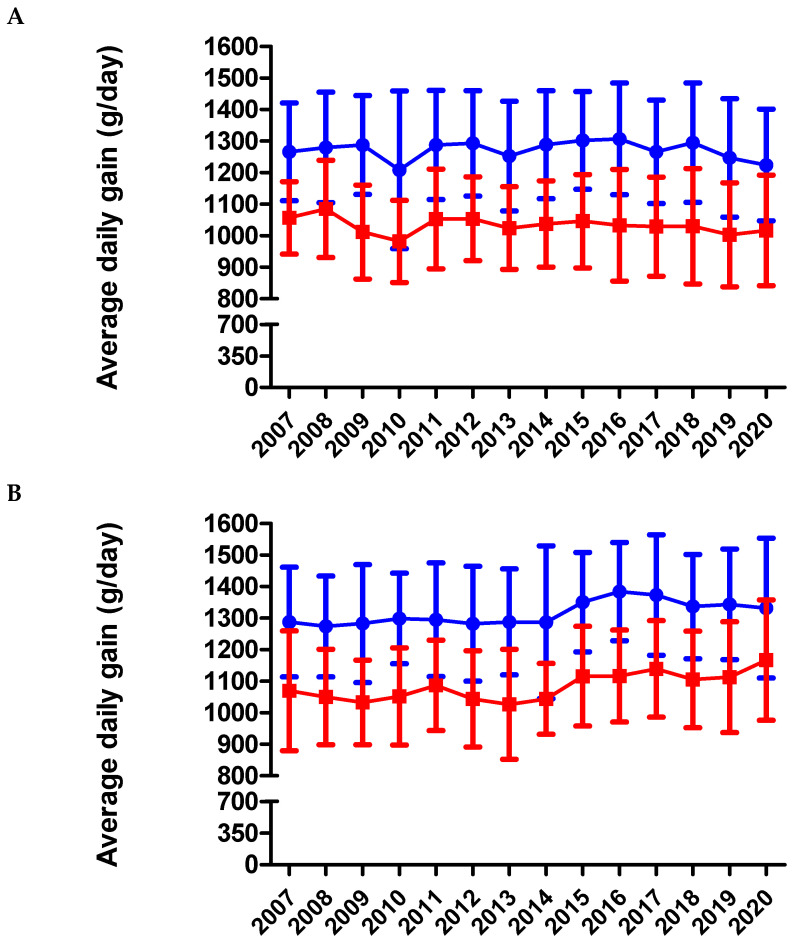
Average daily gain (g/day) from BA (**A**) and BAxL (**B**) split by males (blue filled circles) and females (red filled squares) from 2007 to 2020 in Andorra. Both genders of the BAxL crossbreed improved regarding ADG across the years (*p* < 0.001) and had a better performance during the fattening process than BA animals (*p* < 0.001). However, both genders of the BA pure breed showed a slight but significant decrease in ADG across the years (*p* < 0.05).

**Figure 11:**
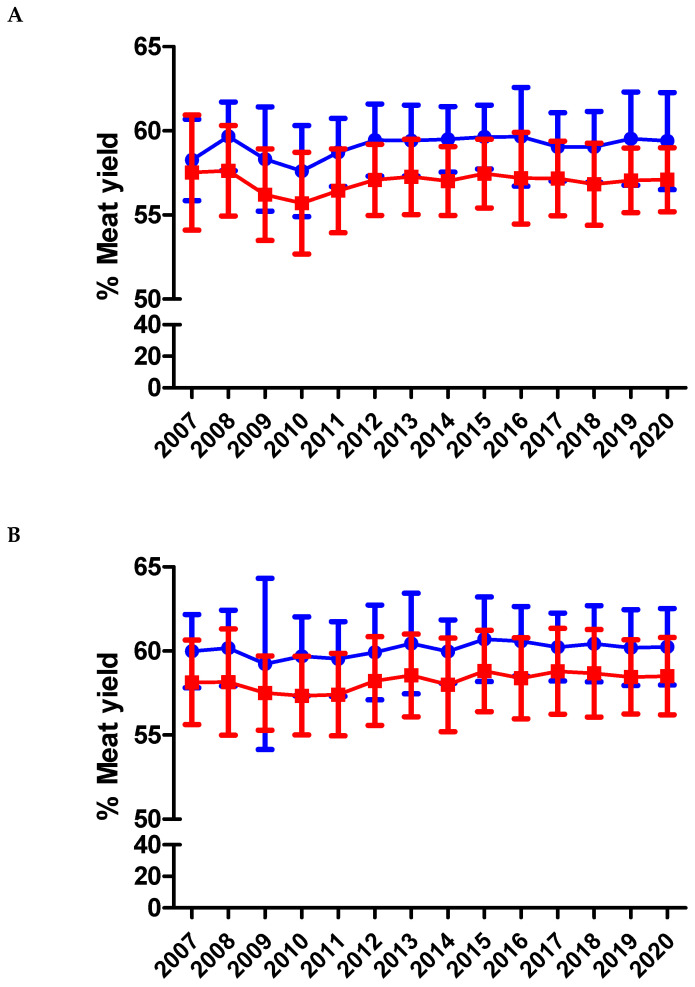
Percentage of meat yield in slaughtered calves from BA (**A**) and BAxL (**B**) split by males (filled circles) and females (filled squares) from 2007 to 2020 in Andorra. Both genders of BAxL crossbreed improved across the years regarding to meat yield (*p* < 0.001) whereas this parameter does not show any significant change across the years for both genders of BA (*p* > 0.05).

Both genders of the BAxL crossbreed showed a significant improvement along the period regarding the ADG and meat yield (*p* < 0.001) and had better performance during the fattening process than BA animals (*p* < 0.001) (Figure 10A,B). On average, BA males had an ADG of 1277 ± 175 and females 1031 ± 156 g/day. In BAxL males, the ADG was 1305 ± 175 g/day and BAxL females averaged 1070 ± 152 g/day (see Table S2 in Supplementary Materials for yearly detailed results). In contrast, both genders of the BA pure breed showed a slight but significant decrease (*p* < 0.05) of ADG along the period. This decrease in BA ADG might be explained because the project priority was the maternal characteristics and a better calving ease for the pure breed. The fact that discarded animals as breeders have been finally slaughtered at older ages might also penalize the BA ADG. The ADG in pure BA are lower than those reported in similar breeds such as BP, where 74 BP bulls had an ADG of 1630 g/day [5] or PA, officially reporting 1750 g/day in males during the fattening period [12]. One Spanish study, with 60 G males under a fattening intensive system and slaughtered at 15 months reported 1300–1520 g/day [27]. Another study also carried out in Spain and under intensive fattening systems, with approximately 80 bulls per breed studied, also reported higher ADG for G (1370 g/day), A (1250 g/day) and S (1290 g/day) than BA males’ ADG [4]. BAxL males averaged 60.03 ± 2.33% of meat yield while BA males never reached 60% (59.24 ± 2.19%). In females, BAxL had 58.08 ± 2.49% while BA never reached 58% (57.05 ± 2.30%) (Figure 11A,B). Although these were slightly lower percentages than similar breeds adapted to the pasture-based mountain areas such as BP, G or A [4,5,6,27] and very similar with S breed (58.5%) [4], this result seems logical because the priority of the BA genetic program is maternal aptitude more than meat performance. It must be highlighted that comparison with other studies should be carried out carefully because fattening systems and age at slaughter can be different and affect the parameters under study.

The carcass quality of the slaughtered animals was monitored with the SEUROP score and began in 2007. No significant differences have been observed between females and males or between BA and BAxL across the study. Since 2009, the percentage of animals classified as ≥R score has been over 95%. Of special interest is to remark the rapid increase in the percentage of BA animals with ≥R score. In 2007, there was only >75% with ≥R score in both BA females and males and this value has reached >95% since 2009. On the other hand, L pure breed animals usually record >95% with ≥U score [25]. Thus, a better carcass quality would be expected for BAxL versus BA animals, but this is not the case. There is the possibility that the crossbreeding with a less performed breed such as BA, the voluntary avoidance of the muscular hypertrophy gene, and the Andorran harsh conditions reduce the carcass quality of the BAxL compared to the pure L carcass and meat quality are strongly affected not only by the breed, but also by the production system [26,28]. Thus, comparison with other studies is difficult because breed characteristics and production systems are different. However, the fact that the PA official site [12] reports E and U as the most common carcass classification should indicate that there is margin to improve carcass quality in BA.

### 3.3. Calving Ease and Reproductive Characteristics of the Breed

Since the start of the program, calving ease was one of the most important parameters to control. The proportion of dystocia has been continuously below 8% (scores CE3 and 4) during the whole period under study, except in 2009 when it was 12.51%. We did not find any reason to explain this punctual increase in dystocia (see Table S2 in Supplementary Materials, available data since 2008) and is in accordance with similar local or European breeds adapted to similar conditions [22,24,29]. This goal has been achieved because the genetic program always considered a better maternal aptitude of the cows (anatomy and behavior), the eradication of muscle hypertrophy (Mh/Mh) in BA bulls, and a reduction of BW at birth (Table S2 in Supplementary Materials). The proportion of bulls that are not carriers for Mh (+/+) has been increasing across the years, from 66.6% (6/9) in the year 2000 to 96.3% (52/54) in 2020 (see Table S1 in Supplementary Materials for yearly data). Thus, farmers increased the use of BA bulls in substitution of L bulls for mating heifers without any negative impact on calving ease. Although the eradication of Mh in the BA breed may penalize in terms of fattening efficiency (lower meat yield and carcass quality) [30], the main goal of the genetic program was to increase the number of BA breeders in order to avoid inbreeding and to have a breed very well adapted to a high mountainous grazing area with limited resources and harsh conditions (climatology and altitude). Although the database provides calving ease scores since 2005, scoring began being systematically recorded in 2008. Since then, the average percent of dystocia (scores CE3 and 4) has significantly dropped from 7% at the beginning of the project (2007–2011) to <3% for the last five years (2016–2020) (*p* < 0.001).

The age at first calving, the average number of parturitions per cow and interval calving–calving were also studied in order to evaluate reproduction performance [31] and decipher if some of these parameters could improve the system and make it more efficient. The average age at first calving was 35.6 ± 5.8 months of age, the average number of parturitions was 4.47 ± 3.08, and interval calving–calving was 13.8 ± 3.9 months. These values are slightly higher but in accordance than the ones reported in similar local breeds, such as BP or PA with ~33 and 32 months at first calving or ~13 and 12.8 months of interval calving–calving, respectively [12,16,32]. The value for the age at first calving in other European breeds, adapted to pastures in the Pyrenees, such as L (33.7 months) or G (31–34 months) was lower than the valued described for BA. [7,33]. While there were no significant differences between years, the three parameters tended to improve (Table S2 in Supplementary Material). We believe that we must be very careful in attributing changes in these parameters to BA cows and their genetic improvement, as a very important limiting factor is the ancestral opinion of farmers, who have always set an age in the first parturition of a cow at 36 months. We consider that a decrease in the age at first parturition should be set as a goal for the next future without any supplementary risk of dystocia, since very similar breeds set 30–33 months of age as a gold standard [12,16,32]. The reduction of the age at first parturition would help in maximizing the efficiency of the Andorran cow–calf farming system.

### 3.4. Slaughter and Meat Products

From the beginning of the project until today, Andorra’s national slaughterhouse has been the only slaughterhouse authorized to slaughter cattle with the label CQCA/IGO Carn d’Andorra. 

The distribution and marketing of meat has varied over the years. Initially, meat sales went through a meat wholesaler who managed sales in both butchers and restaurants (2000–2010). Afterwards, RASA decided to directly distribute the meat to butchers and restaurants. During the first 10 years of the project (2000–2010), the number of butchers and restaurants that sold CQCA products was variable and the authors of this work have not been able to obtain exact data. During the last ten years, RASA directly distributes the meat to 20 butchers (six of them located in supermarkets), three secondary meat distributors, three catering centers, one hospital, one school, and a health center. Since 2015, one of the secondary distributors (Poltrand-www.poltrand.ad. Accessed on 29 September 2020) has also been offering direct online sales of CQCA products. In an effort to maximize the consumption of CQCA products and maximize the sustainability of the production system, RASA is making many efforts to promote the consumption of parts of the carcass considered second category in the commercial circuit. This promotion is carried out with the incorporation of a butcher and a cook in charge of explaining and promoting ways of consuming and cooking these second category meat and viscera pieces.

## 4. Conclusions

Pasture-based livestock farming with local breeds can generate income in regions with limited resources and harsh conditions, and is a key point to the biodiversity conservation of the area. The project of the BA cow breed has shown that it is possible to bet on local productive systems adapted to the natural resources of the environment. The products obtained from these production systems can take place in a local market where farmers and consumers are able to appreciate the values of the cow–calf grazing system with a local breed, even if this means worse production parameters, meat yield, or a higher price compared to other farming and marketing systems. A low inbreeding risk, population, and maternal aptitude in the BA breed seem guaranteed. The BA breed can, and should, make efforts in order to improve the age at first calving, fattening efficiency, and carcass quality. However, we believe that these projects need long-term governmental and private cooperation in all the areas to achieve the goals and be successful.

## Supplementary Materials

The following are available online at https://www.mdpi.com/2076-2615/11/3/611/s1, Table S1: Descriptive results of the population of Bruna d’Andorra (BA) and Bruna d’Andorra crossbreed with Limousin (BaxL) during the last twenty-one years (2000–2020), Table S2: Reproductive and productive data of Bruna d’Andorra (BA) and Bruna d’Andorra crossbreed with Limousin (BaxL) population during the last twenty-one years (2000–2020).
