# Supplementary material for: A Cow–Calf Farming System Fully Adapted to Elevation and Harsh Conditions in Andorra (Europe)
Source: Animals (Basel). 2021 Feb 26;11(3):611. doi: 10.3390/ani11030611 (PMC7996773; doi:10.3390/ani11030611)
Supplement: Supplementary file 1 [file animals-11-00611-s001.pdf]

Table 1.- Descriptive results of the population of Bruna d'Andorra (BA) and Bruna d'Andorra crossbreed with Limousin (BaxL) during the last twenty-one years (2000-2020).

		Year																				
		2000	2001	2002	2003	2004	2005	2006	2007	2008	2009	2010	2011	2012	2013	2014	2015	2016	2017	2018	2019	2020
Adult Cattle																						
	Females older 12 months (n)	943	971	1037	993	1150	1143	1267	1235	1291	1313	1333	1323	1220	1311	1299	1261	1127	1296	1324	1161	1206
	BA Females >12 months (n)	384	411	427	439	795	839	952	958	1027	1061	1111	1115	1058	1152	1165	1148	1038	1202	1237	1102	1149
	Proportion BA/Total (%)	40.7	42.3	41.2	44.2	69.1	73.4	75.1	77.6	79.5	80.7	83.3	84.3	86.7	87.9	89.7	91.0	92.1	92.7	93.4	94.9	95.4
	BA Bulls >12 months (n)	9	16	24	37	41	34	40	39	45	38	37	49	49	54	57	50	62	66	60	61	54
	+/+	6	8	13	19	23	19	24	26	33	31	34	44	46	47	47	44	52	56	54	56	52
	Mh/+	3	8	11	18	18	15	16	13	12	7	3	5	3	7	10	6	10	10	6	5	2
	Limousin Bulls >12 months (n)	17	21	18	19	25	24	17	27	23	13	10	11	11	9	9	8	8	7	6	1	1
Born (n)																						
	BA females	149	104	136	121	105	163	142	181	271	160	266	320	396	372	420	396	412	381	376	362	373
	BAxL females	54	92	122	162	165	108	95	64	93	115	100	103	88	81	47	74	56	104	83	111	101
	BA males	5	9	11	17	16	33	40	78	241	82	248	311	383	368	426	385	386	366	384	341	357
	BAxL males	75	95	111	125	180	128	87	83	97	124	82	103	76	45	47	66	77	89	95	120	112
Slaughter (n)																						
	BA females	0	0	0	0	5	22	¿??	9	37	35	31	125	172	246	227	197	173	246	237	206	227
	BAxL females	39	38	82	108	127	160	148	151	127	137	174	133	131	106	77	41	60	81	80	78	87
	BA males	0	1	4	0	5	7	3	20	22	21	31	220	256	320	345	331	355	333	302	319	339
	BAxL males	36	57	89	111	108	165	153	157	143	172	176	126	123	99	72	58	91	90	77	94	91

BA: Bruna d'Andorra breed.

BaxL: Crossbreed Bruna d'Andorra x Limousin

+/+: Negative to Muscular hypertrophy gene.

Mh/+: Carrier of Muscular hypertrophy gene.

Table 2. Reproductive and productive data of Bruna d'Andorra (BA) and Bruna d'Andorra crossbreed with Limousin (BaxL) population during the last twenty-one years (2000-2020).

		Year																				
		2000	2001	2002	2003	2004	2005	2006	2007	2008	2009	2010	2011	2012	2013	2014	2015	2016	2017	2018	2019	2020
Age at 1st Parturition (months)		34.72±7.31	34.32±4.75	34.21±7.77	36.60±4.52	35.21±5.44	34.64±5.68	35.22±5.46	33.63±5.65	33.37±5.97	35.98±5.92	33.90±3.91	35.49±4.96	33.96±3.93	34.35±2.72	33.92±3.17	33.80±3.74	33.07±3.90	33.37±3.57	33.69±3.89	33.85±4.01	34.71±[illegible]
Average Number Parturition (n)		4.87±2.66	4.01±2.95	3.88±2.56	4.07±3.09	3.74±2.70	4.00±3.17	4.34±3.43	4.29±3.19	4.37±3.04	4.64±2.90	4.47±3.16	4.34±3.02	4.44±3.12	4.37±3.07	4.26±3.02	4.85±3.21	4.25±2.93	4.20±3.21	4.52±3.11	4.36±3.09	4.5±3.[illegible]
Calving Interval (months)		15.01±9.72	15.63±10.21	15.72±9.03	15.09±12.92	15.35±9.01	15.07±7.87	15.05±7.41	15.49±7.72	14.15±5.84	15.48±8.99	13.74±5.90	13.83±6.83	14.19±5.00	14.43±5.92	14.95±5.66	14.42±6.20	14.13±5.71	13.90±4.62	14.33±5.20	14.43±5.08	14.44±[illegible]
Dystocia (scores CE3 and 4)(%)		-	-	-	-	-	-	-	-	7.69	12.51	1.69	4.88	5.20	2.54	2.42	4.24	3.27	3.57	3.03	2.94	2.99
Birth BW (kg)																						
Females	BA	-	-	-	-	-	42.36±5.79	41.85±6.27	42.77±7.49	41.88±6.48	41.72±7.01	41.53±6.84	42.57±7.35	42.03±6.73	42.47±5.93	41.53±6.76	39.86±5.79	37.80±6.81	37.97±7.93	37.05±6.89	38.07±4.19	39.18±[illegible]
	BAxL	-	-	-	-	-	41.89±7.45	41.40±7.10	42.17±6.78	39.97±6.59	41.11±7.85	41.55±6.73	41.61±6.54	41.24±7.02	41.65±6.83	41.09±7.18	40.27±6.12	38.76±8.34	38.82±6.79	38.13±5.82	38.62±6.73	38.87±[illegible]
Males	BA	-	-	-	-	-	43.65±5.79	43.70±4.12	42.87±3.72	42.48±4.49	43.08±5.17	44.68±7.23	44.23±6.21	44.17±4.79	44.31±6.89	43.18±5.73	43.75±6.12	42.55±5.54	41.24±6.32	39.89±4.69	39.56±5.02	40.05±[illegible]
	BAxL	-	-	-	-	-	42.88±6.54	43.79±7.94	44.32±8.37	43.75±2.53	44.41±7.12	42.19±2.57	43.53±6.79	43.37±5.24	42.57±6.45	42.78±5.78	41.35±4.89	39.75±7.98	39.67±5.64	38.29±4.63	39.27±6.12	39.74±[illegible]
Age at slaughter (d)																						
Females	BA	-	-	-	-	435±69	890±45	-	338±48	364±31	390±40	382±70	352±42	368±39	374±44	367±44	360±38	381±69	371±46	371±72	388±55	387±[illegible]
	BAxL	368±37	373±59	348±39	348±46	366±35	370±43	328±17	362±43	372±55	377±42	351±37	351±34	366±42	380±45	366±38	356±40	368±38	347±40	357±34	365±61	352±[illegible]
Males	BA	-	455.00±0	347±41	-	374±58	1057±56	404±64	334±43	368±33	374±32	355±57	354±39	359±36	371±43	359±40	358±38	364±43	366±48	356±47	373±48	374±[illegible]
	BAxL	360±70	342±37	344±33	369±38	353±35	347±41	317±30	353±38	366±34	371±48	353±34	348±39	365±37	371±40	359±41	359±36	361±38	348±50	356±41	355±57	354±[illegible]
BW at Slaughter (kg)																						
Females	BA	-	-	-	-	-	-	-	398.00±38.68	435.46±44.12	433.58±51.34	411.58±39.23	409.10±43.47	427.87±46.49	423.34±47.83	419.65±51.23	414.42±51.28	426.16±63.39	415.76±49.34	410.17±49.93	422.25±52.67	427.10±[illegible]20
	BAxL	-	-	-	-	-	-	371.77±21.38	388.54±30.86	425.32±42.53	428.08±50.10	408.80±47.00	421-20±47.49	421.75±54.36	429.32±60.15	421.64±42.89	434.39±47.81	448.05±50.54	431.12±48.61	432.20±52.27	439.84±57.96	439.31±[illegible]55
Males	BA	-	-	-	-	-	-	571.53±10.78	462.81±53.87	514.02±72.65	523.88±83.21	464.65±63.66	497.30±57.91	506.30±60.18	507.77±63.94	504.03±62.53	507.81±58.09	515.92±62.54	502.46±66.89	496.78±59.72	499.69±57.04	494.73±[illegible]75
	BAxL	-	-	-	-	-	-	412.41±32.37	496.02±54.09	509.33±58.93	509.45±59.46	500.39±55.07	491.09±53.95	508.11±60.90	517.85±60.88	499.11±59.16	525.20±60.92	537.90±56.16	512.44±55.09	512.36±63.59	511.04±52.74	508.59±[illegible]05
ADG (g/d)																						
Females	BA	-	-	-	-	-	-	-	1,057±115	1,085±154	1,012±149	982±130	1,053±158	1,054±133	1,024±131	1,037±137	1,046±148	1,033±177	1,029±157	1,030±183	1,003±165	1,017±[illegible]
	BAxL	-	-	-	-	-	- -	1,084±90	1,070±410	1,050±151	1,033±134	1,052±154	1,087±143	1,044±153	1,027±174	1,044±112	1,116±158	1,117±146	1,139±153	1,106±153	1,113±176	1,167±[illegible]
Males	BA	-	-	-	-	-	-	1,323±236	1,266±155	1,280±175	1,288±157	1,209±250	1,288±173	1,293±167	1,253±174	1,289±171	1,302±155	1,307±177	1,266±164	1,295±189	1,247±188	1,224±[illegible]
	BAxL	-	-	-	-	-	-	1,298±143	1,288±174	1,274±160	1,283±187	1,299±144	1,295±180	1,282±182	1,288±168	1,287±242	1,351±158	1,384±156	1,373±191	1,337±165	1,344±175	1,332±[illegible]
% Carcass/BW																						
Females	BA	-	-	-	-	-	-	-	57.52±3.42	57.63±2.69	56.20±2.72	55.70±3.03	56.44±2.49	57.09±2.11	57.27±2.24	57.02±2.04	57.46±2.05	57.18±2.72	57.17±2.22	56.83±2.44	57.06±1.91	57.33±[illegible]
	BAxL	-	-	-	-	-	-	58.55±2.11	58.14±2.51	58.15±3.16	57.50±2.20	57.34±2.34	57.40±2.45	58.22±2.64	58.55±2.46	57.99±2.78	58.81±2.41	58.38±2.64	58.79±2.55	58.68±2.06	58.46±2.20	58.37±[illegible]
Males	BA	-	-	-	-	-	-	59.37±1.44	58.27±2.42	59.67±2.04	58.32±3.10	57.61±2.71	58.72±2.02	59.45±2.15	59.42±2.11	59.50±1.95	59.63±1.90	59.65±2.93	59.05±2.03	59.04±2.11	59.53±2.77	59.27±[illegible]
	BAxL	-	-	-	-	-	-	59.49±2.10	59.99±2.38	60.17±2.25	59.23±5.08	59.69±2.35	59.53±2.22	59.92±2.81	60.45±2.99	59.97±1.88	60.70±2.52	60.58±2.07	60.23±2.02	60.43±2.26	60.20±2.03	60.16±[illegible]
SEUROP Category (%SEUR)																						
Females	BA	-	-	-	-	-	-	-	>75%	>85%	>95%	>95%	>95%	>95%	>95%	>95%	>95%	>95%	>95%	>95%	>95%	>95%
	BAxL	-	-	-	-	-	-	>95%	>95%	>95%	>95%	>95%	>95%	>95%	>95%	>95%	>95%	>95%	>95%	>95%	>95%	>95%
Males	BA	-	-	-	-	-	-	>75%	>75%	>85%	>95%	>95%	>95%	>95%	>95%	>95%	>95%	>95%	>95%	>95%	>95%	>95%
	BAxL	-	-	-	-	-	-	>95%	>95%	>95%	>95%	>95%	>95%	>95%	>95%	>95%	>95%	>95%	>95%	>95%	>95%	>95%

BA: Bruna d’Andorra breed.

BaxL: Crossbreed Bruna d’Andorra x Limousin

CE: Calving ease.

BW: Body weight

ADG: Average daily gain

SEUROP category: European carcass rating based on muscles profile.
